# Verapamil-associated cardiogenic shock in a 71-year-old man with myasthenia gravis: a case report

**DOI:** 10.4076/1752-1947-3-8219

**Published:** 2009-06-16

**Authors:** Benoit Drolet, Geneviève Gabra, Chantale Simard, Bernard Noël, Paul Poirier

**Affiliations:** 1 Quebec Heart Institute, Laval Hospital, Quebec City, Quebec, Canada; 2 Faculty of Pharmacy, Laval University, Quebec City, Quebec, Canada

## Abstract

**Introduction:**

Myasthenia gravis is a rare neuromuscular disorder associated with a reduction in the availability of acetylcholine receptors at the post-synaptic membranes of skeletal muscles. This is caused by the production of anti-acetylcholine receptor antibodies at the neuromuscular junction due to an autoimmune insult, leading to a compromised neuromuscular transmission. Verapamil can influence, in a dose-dependent fashion, the neuromuscular transmission in myasthenia gravis.

**Case presentation:**

We report a 71-year-old Caucasian man with myasthenia gravis suffering from a cardiogenic shock following a single dose of verapamil. The patient had uncontrolled atrial fibrillation with a heart rate of 120 beats/min. Atenolol 100 mg was started. The next day, verapamil SR 240 mg was started. Two hours after the first dose of verapamil, the patient complained of weakness and dyspnea with signs of shock; his blood pressure was 70/50 mm Hg and heart rate at 101 beats/min. An echocardiogram showed diffuse hypokinesis of both ventricles with an ejection fraction of 20%. Cardiac catheterization was performed and coronary arteries appeared without significant stenosis, but there was a diffuse hypokinesis. Verapamil was stopped and the patient received intravenous glucagon and calcium chloride. Both the anti-acetylcholine receptor and anti-striated muscle antibodies tested positive. A few hours later, another echocardiogram showed an improvement in the ventricular function, which returned to normal five days later.

**Conclusion:**

Caution is needed when administering verapamil to patients with myasthenia gravis, especially when the anti-acetylcholine receptor and anti-striated muscle antibodies titres are positive.

## Introduction

Myasthenia gravis (MG) is a rare (4 cases/100,000 individuals) neuromuscular disorder characterized by weakness and excessive fatigability of skeletal muscles following repetitive effort and slow recovery after exercise [1]. The defect in MG is a reduction in the availability of acetylcholine receptors at the post-synaptic membranes of skeletal muscles. This is caused by the production of anti-acetylcholine receptor antibodies (AchR-Ab) at the neuromuscular junction (NMJ) due to an auto-immune insult [2], leading to compromised neuromuscular transmission (NMT). Verapamil is a calcium channel blocker useful in lowering blood pressure and in slowing cardiac atrioventricular conduction. It can also affect, in a dose-dependent fashion, the NMT in MG [3]. Verapamil's most commonly reported adverse effects are constipation, dizziness and nausea. We describe a patient with known MG who experienced cardiogenic shock following administration of a dose of verapamil.

## Case presentation

**Figure 1:**
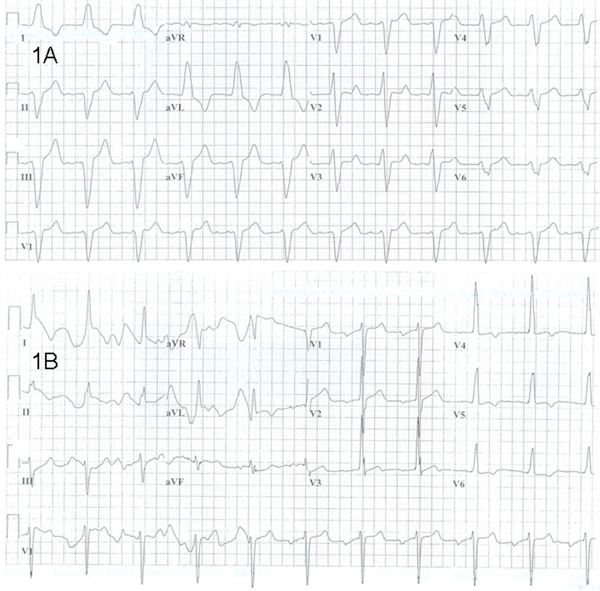
****(A)** 12-lead electrocardiogram in the Myasthenia Gravis patient two hours after the first dose of verapamil SR 240 mg and just a few minutes before cardiogenic shock**. Spectacular widening of the QRS at 230 msec is observed (paper speed: 25 mm/sec). **(B)** 12-lead electrocardiogram in the Myasthenia Gravis patient in the intensive care unit two hours after verapamil withdrawal and intravenous administration of calcium chloride and glucagon. Significant hemodynamic improvement was already observed. QRS narrowing at 138 msec was also seen (paper speed: 25 mm/sec).

A 71-year-old Caucasian male with MG was known to suffer from nonobstructive hypertrophic cardiomyopathy and paroxysmal atrial fibrillation and a dilated left atrium. Multiple unsuccessful electrical and chemical cardioversions (using sotalol and amiodarone) had been attempted in the past. Therefore, ablation of the AV node was performed and a permanent dual chamber pacemaker was implanted. Nonetheless, at the time of admission to hospital the patient was complaining of fatigue, palpitations and dyspnea that were rapidly linked to uncontrolled atrial fibrillation at a heart rate of 120/minute. Previously, for a long time he was on metoprolol 100 mg BID concomitantly with diltiazem 300 mg OD for heart rhythm control. To further control the heart rate, he received in sequence atenolol 100 mg OD and, more than 24 hours after diltiazem cessation, verapamil SR 240 mg OD to replace his previous regimen. Two hours after receiving his first dose of verapamil, the patient began to complain of weakness and dyspnea. He presented with signs of shock with blood pressure at 70/50 mm Hg and heart rate at 101/minute. Lung and cardiac auscultation appeared normal with no new murmur of acute valvular failure, pulmonary congestion or signs of pulmonary embolism. An electrocardiogram (ECG) revealed paced rhythm (Figure 1). A quick bedside echocardiogram showed diffuse hypokinesis of both ventricles with an ejection fraction reduced to 20%. Eighteen months before, an echocardiogram revealed normal left ventricular function and ejection fraction of 66%. At that time, the ventricular rate was 110 beats/min while the patient was on long-acting diltiazem 240 mg OD and cilazapril 5 mg OD. Cardiac catheterization was performed within minutes; coronary arteries appeared without significant stenosis, but there was a diffuse hypokinesis. At this point, cardiogenic shock secondary to calcium channel blocker intoxication was suspected. Blood glucose was 4.3 mmol/L, electrolytes (Na^+^, K^+^, Cl^-^) were 138, 5.2, and 105 mmol/L, respectively, all within normal limits. One hundred percent oxygen administration was performed resulting in a saturation of 98% and a pCO_2_ to 41.5 mm Hg (normal 35 to 45 mm Hg). Verapamil was stopped and the patient received intravenous glucagon, calcium chloride and dopamine. The measure of AchR-Ab was positive at 0.23 nmol/L (normal ≤0.02 nmol/L) and the measurement of antistriated muscle antibodies was also positive at 1:3840 (normal <1:60). A few hours later, another echocardiogram was performed and there was an improvement in ventricular function, resulting in recovery of systolic blood pressure to between 125 and 130 mm Hg. Five days later, the ejection fraction had returned to normal as had as the patient's electrocardiogram (Figure 1). Re-challenge with verapamil was not performed. The patient died two years later.

## Discussion

The histologic striated muscle changes found in MG are fibre atrophy with varying degrees of inflammation [4]. Of importance is the fact that antibodies directed against striated muscle are found in the serum of about one third of MG patients [5]. Table 1 lists numerous classes of drugs that have been reported to exacerbate MG.

**Table 1:** Classes of drugs known to exacerbate Myasthenia Gravis

Antibiotics (aminoglycosides, polypeptides, tetracyclines)
Quinine and related drugs
Antirheumatic drugs
Cardiovascular drugs: beta-blockers, procainamide, quinidine
Anti-epileptics
Benzodiazepines
Neuroleptics
Anaesthetics
Analgesics
Corticosteroids
D-penicillamine
Antihistamines
Diuretics
Anticholinergics

The calcium channel blocker (CCB) verapamil is useful in the treatment of recurrent supraventricular arrhythmias. It blocks the slow calcium ion influx into contractile and conduction fibres, prolongs the refractory period in nodal cells and may also depress contractility [6]. Respiratory failure immediately following intravenous injection of verapamil has been reported in a patient with Duchenne's dystrophy [7]. This suggests that verapamil might exacerbate muscle weakness when NMT is impaired. Drug-induced neuromuscular blockade is uncommon in subjects without MG, presumably because of the high safety margin for neuromuscular transmission that exists under normal circumstances.

Previous studies evaluating the effects of CCBs on the NMT showed that these agents impair transmission in the NMJ, especially in conjunction with a second neuromuscular blocking agent [8]. The safety margin for functional NMT was reported to be as high as 80% to 90% blockade of all post-junction receptors [9]. It was reported that MG muscle showed a 70% to 89% reduction in the number of acetylcholine receptors per neuromuscular junction, compared to the control muscle [10], thus strongly suggesting that verapamil can affect NMT in individuals with MG at subclinical doses. In other words, the deleterious neuromuscular action of verapamil in MG is unmasked by the narrow safety margin resulting in a reduction in "receptor reserve". Similarly, verapamil-induced aggravation of Lambert-Eaton myasthenic syndrome has also been described [11]. This might be a consequence of the effect on voltage-dependent calcium channels involved in the release of acetylcholine at the presynaptic nerve terminal, the probable target of the immune response leading to Lambert-Eaton syndrome (LES) [11].

Indeed, worsening of muscle weakness upon exposure to verapamil has clearly been demonstrated in disorders of NMT such as MG and LES [3]. Moreover, it was suggested that this phenomenon is exacerbated when AchR-Ab and antistriated muscle antibodies are positive. In our patient, the measurement of AchR-Ab was positive at 0.23 nmol/L (normal ≤0.02 nmol/L) and antistriated muscle antibodies were also positive at 1:3840 (normal <1:60); the time course of clinical deterioration pointed to verapamil as the responsible drug. This hypothesis is supported given that not only did the patient begin complaining of weakness and dyspnea within 2 hours of the first and only verapamil dose, but he also presented with signs of cardiogenic shock with blood pressure of 70/50 mm Hg and a heart rate of 101 beat/min. *A posteriori*, verapamil appears as the likely "missing link" between these apparently unrelated symptoms. Indeed, bradycardia, transient asystole and exacerbation of heart failure have been reported with verapamil, although these responses usually occurred after intravenous administration of the drug or in the presence of β-adrenergic receptor blockade [6,12]. In this case, oral verapamil was added to atenolol, potentially contributing to further acute myocardial hemodynamic depression, as it has been frequently reported in the past [6,12]. However, if further cardiac deterioration upon exposure to verapamil was solely due to its calcium channel blocking properties, it should have been observed before, while the patient was on a combined metoprolol and diltiazem regimen. In fact, in this case, a few minutes before the cardiogenic shock, a pacemaker-induced sinus rhythm was recorded and spectacular widening of the QRS (230 msec) was observed (Figure 1A), suggesting further deterioration of cardiac conduction upon exposure to verapamil. Interestingly, in addition to its calcium channel-blocking properties, and unlike diltiazem, verapamil has been reported to block both the fast and late cardiac sodium currents [13,14]. In a patient such as ours, for whom calcium-dependent depolarization is compromised, conduction capacity strongly relies on the cardiac sodium current (I_Na_). It is therefore likely that verapamil, with its I_Na_-blocking effect, depleted the "vital conduction reserve" of this patient, leading to cardiac decompensation and shock. It is noteworthy that this has never been observed with diltiazem before due to lack of I_Na_-blocking influence. QRS narrowing following verapamil withdrawal and administration of calcium chloride and glucagon (Figure 1B) is also consistent with this explanation. Nevertheless, one could argue that diltiazem is also a CCB known to affect the NMT. However, our patient had been treated safely with this drug, well before the onset of the cardiac deterioration. Moreover, as the elimination half-life of diltiazem is estimated at 5 hours, and as verapamil was initiated more than 24 hours after diltiazem cessation, approximately 5 half-lives, it appears unlikely that a significant combined calcium channel blocking action of both drugs was responsible for the patient's neuromuscular and cardiac deterioration.

Another possible explanation for the clinically observed rapid hemodynamic deterioration of this patient was the development of Takotsubo syndrome. Takotsubo cardiomyopathy, also known as transient apical ballooning or apical ballooning cardiomyopathy, is a type of nonischemic cardiomyopathy in which there is a sudden temporary weakening of the myocardium [15]. The typical presentation of someone with Takotsubo cardiomyopathy is sudden onset of congestive heart failure or chest pain associated with ECG changes suggestive of an anterior wall heart attack. During the course of evaluation of the patient, a bulging out of the left ventricular apex with a normo- or hypercontractile base of the left ventricle is considered the hallmark of this syndrome. Indeed, the diagnosis is made by the pathognomic wall motion abnormalities, in which the base of the left ventricle is contracting normally or is hyperkinetic while the remainder of the left ventricle is akinetic or dyskinetic. This is accompanied by the lack of significant coronary artery disease that could explain the wall motion abnormalities. Provided that the individual survives the initial presentation, the left ventricular function improves within 2 months.

An argument against the Takotsubo syndrome hypothesis in this case is the fact that hypokinesis was shown to be diffuse and in both ventricles. Bulging of the apex was never observed. Moreover, only a few hours after verapamil withdrawal, ventricular function improved. Five days later, the ejection fraction returned to normal as well as the patient's electrocardiogram.

## Conclusion

We described a patient with known MG with cardiogenic shock following administration of verapamil, a drug known to cause a deleterious impact on the NMT. This case strongly suggests that caution is needed when administering verapamil or other CCBs to MG patients with impaired NMT, especially when AchR-Ab and the antistriated muscle antibodies titres are positive.

## Abbreviations

AChR-Ab: Acetylcholine receptor-antibodies; BID: Twice daily; CCB: Calcium channel blocker; ECG: electrocardiogram; I_Na_: cardiac sodium current; LES: Lambert-Eaton Syndrome; MG: Myasthenia Gravis; NMJ: Neuromuscular junction; NMT: Neuromuscular transmission; OD: Once daily.

## Consent

Written informed consent was obtained from the patient's wife for publication of this case report and any accompanying images. A copy of the written consent is available for review by the Editor-in-Chief of this journal.

## Competing interests

The authors declare that they have no competing interests.

## Authors' contributions

BD was a major contributor in writing the manuscript. GG analyzed and interpreted the patient's data relative to MG symptoms and contributed to writing and review of the manuscript. CS analyzed the pharmacological profile and medical record of the patient and contributed to writing and review the manuscript. BN is a cardiologist who took care of the patient in the intensive care unit and contributed to writing and review of the manuscript. PP was the cardiologist in charge who dealt with the acute cardiac decompensation of the patient and was a major contributor in writing the manuscript.
